# Frequency and severity of aggressive incidents in acute psychiatric wards in Switzerland

**DOI:** 10.1186/1745-0179-3-30

**Published:** 2007-12-04

**Authors:** Christoph Abderhalden, Ian Needham, Theo Dassen, Ruud Halfens, Joachim E Fischer, Hans-Joachim Haug

**Affiliations:** 1 Nursing and Social Education Research Unit, University Bern Psychiatric Services, Berne, Switzerland; 2 University of Applied Sciences, St. Gallen, Switzerland; 3 Department of Nursing Science, Humboldt-University, Berlin, Germany; 4 Universiteit Maastricht, The Netherlands; 5 Department of Public Health, Social and Preventive Medicine, Mannheim Medical Faculty, University of Heidelberg, Mannheim, Germany; 6 University Zurich and, Psychiatric Hospital Schloessli, Oetwil am See, Switzerland

## Abstract

**Background:**

Aggression and violence and negative consequences thereof are a major concern in acute psychiatric inpatient care globally. Variations in study designs, settings, populations, and data collection methods render comparisons of the incidence of aggressive behaviour in high risk settings difficult.

**Objective:**

To describe the frequency and severity of aggressive incidents in acute psychiatric wards in the German speaking part of Switzerland.

**Methods:**

We conducted a prospective multicentre study on 24 acute admission wards in 12 psychiatric hospitals in the German speaking part of Switzerland. Aggressive incidents were recorded by the revised Staff Observation Aggression Scale (SOAS-R) and we checked the data collection for underreporting. Our sample comprised 2344 treatment episodes of 2017 patients and a total of 41'560 treatment days.

**Results:**

A total of 760 aggressive incidents were registered. We found incidence rates per 100 treatment days between 0.60 (95% CI 0.10–1.78) for physical attacks and 1.83 (1.70–1.97) for all aggressive incidents (including purely verbal aggression). The mean severity was 8.80 ± 4.88 points on the 22-point SOAS-R-severity measure; 46% of the purely verbally aggression was classified as severe (≥ 9 pts.). 53% of the aggressive incidents were followed by a coercive measure, mostly seclusion or seclusion accompanied by medication. In 13% of the patients, one ore more incidents were registered, and 6.9% of the patients were involved in one ore more physical attack. Involuntary admission (OR 2.2; 1.6–2.9), longer length of stay (OR 2.7; 2.0–3.8), and a diagnosis of schizophrenia (ICH-10 F2) (OR 2.1; 1.5–2.9) was associated with a higher risk for aggressive incidents, but no such association was found for age and gender. 38% of the incidents were registered within the first 7 days after admission.

**Conclusion:**

Aggressive incidents in acute admission wards are a frequent and serious problem. Due to the study design we consider the incidence rates as robust and representative for acute wards in German speaking Switzerland, and thus useful as reference for comparative and interventional research. Implications for clinical practice include the recommendation to extend the systematic risk assessment beyond the first days after admission. The study confirms the necessity to differentiate between types of aggressive behaviour when reporting and comparing incidence-data.

## Background

Aggression and violence and negative consequences thereof for health, safety and wellbeing of patients and personal involved are a major concern in inpatient psychiatric care globally. Thus, the reduction of the incidence of aggression and violence and resultant negative effects is a challenge for researchers and staff of psychiatric facilities alike. This issue has led to a voluminous body of research on the incidence of aggression, causes, risk factors, and ways of effective management of aggression among psychiatric patients. In spite of a substantial body of research on the topic considerable variation regarding study aims, study designs, settings, study populations, and data collection methods render comparisons difficult and the results of these studies cannot be summarised easily due to the many disparities in the findings [1-3]. Disturbingly, this is also true for research on basic questions such as the incidence of aggressive behaviour in high risk settings (e.g. acute admission wards).

Some of the most important factors hindering the generalisability and comparability of incidence studies are differing definitions of aggressive behaviour, the variety of registration methods, inconsistent ways of reporting incidence rates, a substantial amount of underreporting, and problems arising from the selection of study settings and the duration of observations.

Due to its multidimensional nature, there is no uniform and purpose-for all-definition of aggression and it its perception is multi-facetted [4,5]. Palmstierna and Wistedt suggested to look at the phenomenon using the dimensions *"inner experience vs. outward behaviour; aggressor's view vs. observer's view; and persistent state vs. episodical occurrence" *[[1], p. 79]. Aggression may be defined as psychological state or as hostile physical or verbal act or as behaviour resulting in injuries of persons or damage to objects. In a similar display of variability conceptions of assault may range from verbal and physical behaviour to sexual harassment. Given such variability a clear cut-off for the severity of violent incidents under investigation [3] is of primary importance, and frequencies should be reported separately for different types of aggression.

Aggressive behaviour can be registered in many different ways, and there are dozens of instruments available [6]. Reviewing the literature on registration methods, Gothelf et al [7] found that almost half of the 103 studies under review did not use any structured instrument; and the remainder used a total of 52 different instruments. Such findings led to calls for the use of more standardised procedures to record violence [1]. In addition Bowers [8], reviewing some of the most frequently used instruments, pointed to some weaknesses of most of these instruments, including the classification of a wide variety of behaviours such as aggression directed towards self and aggression against others into the one category "aggressive incident".

Of special concern are the various ways of reporting incidence rates. As Bowers stated in a review critically appraising this topic *"the expression of ward incident rates has been unclear and disorganised, resulting in incomparability between studies and lack of precision" *[[2], p. 365]. In some studies, the incidence of aggression is approached from the perspective of staff as victims with incidence expressed in terms of the assault rates among populations of nurses or doctors. Other studies focus on the proportion of patients showing assaultive behaviour or on differing types of incidence-rates (e.g. per bed per year, per 100 treatment days etc.). As a result, in one study [9] 0.00069 violent incidents per treatment day were observed, and another study [10] reports 16 injuries per 100 staff (per annum?). Yet other authors found that 13.7% [11] or 2.7% of hospitalised patients were aggressive [12]. In a German study encompassing 162 patients among 9216 admissions aggressive incidents were registered producing an incidence rate of 0.019 equalling 2% of admissions [13], while in two single centre studies conducted at the Psychiatric University Clinic in Zurich 10% of newly admitted patients were aggressive [14,15]. However, in a recent overview on studies all using the same instrument, the authors were able to derive the annual frequency of incidents per bed per year and the percentage of patients involved in incidents as appropriative comparative figures in most of the studies. For acute wards, the respective figures ranged from 1.7 to 31.2 and 6.2% to 45% [16].

Another major obstacle to obtain comparable data on the incidence of aggression or violence in health care settings is underreporting [3,17-20]. Underreporting not only distorts the statistics, but also renders some forms of aggression – especially aggression of a less severe nature – invisible. Lion et al. [18] estimated that there were five times as many assaults as formally reported in their hospital. However, it has been suggested that more severe incidents are more likely to be reported than incidents of less severe nature [21].

One repeatedly drawn conclusion from existing research is to consider specificities of certain settings and to be wary on aggregating data from different populations (e.g. ward types) and different types of aggressive behaviour for research [22,23]. This can be illustrated by studies comparing the incidence of different forms of aggression or the incidence among different settings. Kay et al [24] found a proportion of physically aggressive patients of 26.3% in a secure care unit, compared with 8.7% in an admission ward and 1.4% in a chronic care unit, and the respective figures reported by Miller et al [22] were 27% for a locked short stay ward and 10% for a open short stay ward. The average monthly rate of violent behaviour (number of violent incidents divided by average census) in 13 wards ranged from 0.025 in an adult transitional program to 0.576 in a female acute admissions unit [25]. Another reason to focus on special types of wards is the need for baseline data suitable for the evaluation of setting-adjusted interventions introduced to reduce violence. Findings that general risk factors for violence may not to be useful in acute admission settings underscore the importance of taking setting specific aspects into consideration [26,27].

The generalisability of existing research specifically focusing on acute wards is hampered by the fact that the majority of the studies are single-centre studies, often restricted to one single ward, and – relatively to the incidence – cover a short period of observation and few treatment days. The largest sample is 7 acute wards in 4 hospitals [28], five studies comprise more than 10'000 treatment days [11,28-31]. One of the findings in most studies is a small proportion of patients being responsible for a substantive proportion of incidents. These outliers make the interpretation of rates derived from single wards or short observation periods difficult.

In order to support the international collaboration in improving the management of aggressive behaviour it is imperative to obtain information which allows comparisons of services and caring approaches within health systems and between countries [1,8].

To date only a few studies on aggression among psychiatric inpatients in Switzerland exist. These studies compare characteristics of aggressive and non-aggressive patients [14,15] or focus on the description of incidents [32] or on costs of assaults on personnel [33]. The studies include various types of wards within one hospital [14,15] or all incidents in all wards in a group of six hospitals [34,32], however none of them has investigated the situation of acute admission wards. Only Kaision et al [33] used an internationally widely used instrument (OAS, [35]), while the other studies employed different versions of an own instrument, though showing some similarities to the SOAS. None of these Swiss studies provides incident rates for different forms of aggressive behaviour per treatment day or per bed and year.

### Aim of the study

The aim of this study is to describe the frequency and severity of aggressive incidents in acute psychiatric wards in the German speaking part of Switzerland. Secondary aims are to estimate the extent of underreporting and to explore associations of patients' characteristics with the occurrence of aggressive incidents. The study design aims to meet some of the methodological problems of existing studies.

## Method and materials

We conducted a prospective multicentre study on acute admission wards in the German speaking part of Switzerland. The 32 psychiatric hospitals in this language area provide psychiatric inpatient treatment for approximately 75% (5'376'800 persons) of the Swiss population. Of the 324 wards within these hospitals, 87 are acute wards having the following characteristics: The majority of patients have an acute psychiatric disorder, they are admitted voluntarily or against their will directly onto the ward, they stay less than three months on the ward, they are generally older than 18 and younger than 65 years, and the ward is not specialised in the treatment of special disorders (e.g. depression, addiction). Ten of these wards have a majority of private patients and few involuntarily admitted patients, 7 of them being located within private hospitals without obligation to treat patients from the region they are situated in.

### Setting and sample

After approaching all 87 acute psychiatric wards in the study area 24 (27.6%) wards from 12 hospitals agreed to participate in the study.

### Instruments

Before embarking on this study a survey of all wards within the study area using a questionnaire covering data on the size of the wards, staffing, the facilities for managing aggression and violence [36] was conducted. Additionally, staff nurses were asked to rate the severity of the problem and the resources for aggression management.

Aggressive incidents were recorded by the revised Staff Observation Aggression Scale (SOAS-R) [37]. The SOAS-R covers provoking factors, the means used by the patient, the target of aggression, the consequence for the target, and the measures to terminate the aggression. The scale is to be completed by staff members witnessing aggressive behaviour of a patient whereby aggression is defined as any verbal, non-verbal, or physical behaviour that was threatening (to self, others, or property), or physical behaviour that actually did harm (to self, others, or property) [38]. The severity of the incidents was measured using the SOAS-R-scoring system which ranges from 0 to 22 points, and additionally by a 100-mm-Visual Analogue Scale (VAS) with the endpoints "not severe incident" and "very severe incident".

Data on patients were taken from the hospitals data bases and included dates of admission and discharge, age, gender, and main psychiatric diagnosis according to ICD-10.

In order to estimate possible underreporting visits to most of the study wards were conducted at randomly selected dates. We investigated the written shift reports of the previous three days and noted all descriptions of aggressive incidents. After termination of the study period, we controlled these incident data of the respective wards for SOAS-R-data covering these events, and estimated the severity of the incidents in cases where SOAS-R-forms were missing.

### Data analysis

We included all aggressive incidents directed towards other persons or objects, but excluded purely auto-aggressive incidents. As recommended by the authors of the SOAS-R, events with a severity of 9 or more points on the SOAS-R-severity score were regarded as severe incidents (Henk Nijman, personal communication). Physical attacks were defined as recordings on the SOAS-R fulfilling the two criteria 1) the means of aggression = objects OR dangerous objects OR parts of the body AND 2) the target of the aggression = a person other than the patient her- or himself. We calculated incidence rates for all aggressive incidents, for severe incidents, for physical attacks, physical aggression and purely verbally aggressive incidents. Incident rates were expressed as the proportion of patients involved in aggressive events, as event-rates per 100 hospitalisation days with 95%-confidence-intervals, as event rates per bed per annum, and as the proportion of days with one or more event. In cases of multiple treatment episodes of patients in the study period, we used the first episode as index episode for the patient related analyses.

We employed binary logistic regression to test for associations between patients' characteristics and the incidence. We used the presence or absence of at least one more severe aggressive incident as binary outcome measure. We calculated crude odds ratios (OR) for each of the independent variables separately.

Statistical significance was determined using the traditional cut-off level α = 0.05. We used SPSS (Version 10.0, SPSS, Chicago, IL) and CIA (Confidence Interval Analysis, Version 2.1, University of Southampton, UK) for statistical analysis.

## Results

### Wards

Information on ward characteristics were obtained from 82 of the 87 wards the study area. Twenty four wards with a total of 388 beds participated, representing 12 out of the 32 hospitals and 25% of all acute wards and 28% of all acute beds in the German speaking region of Switzerland. The number of beds per ward ranges from 9 to 19 with a mean number of beds of 16; the mean staff-patient-ratio was .77 Full Time Equivalents (FTE) per bed. Fifteen (62%) of the wards are always closed, 9 wards part of the time, and 22 of the 24 wards have at least one seclusion room. According to the ratings of the staff nurses aggression is deemed a large or very large problem on 13 (54%), as a small or medium problem on 11 (46%) wards. None of the ward nurses rated aggression as very small or no problem at all. Seven (29%) of the wards regarded their resources for managing aggression as insufficient. No significant differences were observed between participating and non-participating wards regarding the number of beds and staff-patient-ratios. The participating wards include a higher proportion of closed wards and of wards having seclusion rooms, wards rating aggression as a problem and the rating their resources for aggression management as insufficient (table 1).

**Table 1:** Ward characteristics

	Participating wards (N = 24)	Non-participating wards (N = 58)*
Number of Beds mean (SD)	16.1 (± 2.4)	16.8 (± 4.1)
Nursing staff (FTE) per bed	0.77 (± 2.2)	0.72 (± 2.4)
Proportion of wards always closed	15 (62.5%)	28 (30.4%)
Proportion of wards with ≥ seclusion room	22 (95.7%)	42 (80.8%)
Aggression rated as		
- no/very small problem	0 (.0%))	10 (17.2%
- small or medium problem	11 (45.8%)	31 (53.4%)
- big/very big problem	13 (54.2%)	17 (29.3%)
Resources for aggression-management rated as		
- sufficient	17 (70.8%)	47 (81.0%)
- unsufficient	17 (29.2%)	11 (19.0%)

* missing information on 5 of the 87 non-participating wards

### Observation period

The observation period was three month per ward. The study period covered 72 month (24 wards × 3 month) and included all seasons of the year: 13 month of observation in January-March, 5 in April-June, 16 in July-September, and 38 in October – December.

### Patients

During the study period 2017 patients (46.6% females, mean age 39.4 ± 13.9 years, range 12 – 96 years) accounted for 2344 treatment episodes on the 24 study wards. The hospitalisations comprised of 60.9% voluntary and 39.1% involuntary admissions giving rise to a total of 41'560 treatment days. The length of the treatment episodes ranged between one and 133 days (median 9, Mean 17.7 ± 22.0 days). The patient's ICD-10 diagnoses comprised of schizophrenia, schizo-type and delusional disorders (ICD F2: 29.3%), mood (affective) disorders (F3: 16.7%), mental and behavioural disorders due to psychoactive substance use (F1: 24.9%), neurosis (F4: 13.7%) and personality disorders (F6: 4.1%), and other ICD-10 categories (F0, F5, F7, F8, F9, others: 3.4%) (4.7% missing).

### Aggressive incidents

A total of 760 aggressive incidents were reported, including 396 incidents with a SOAS-R severity score of 9 or more and 252 physical attacks (the latter do not necessarily score 9 or more). 157 incidents were purely verbal aggression. These figures correspond to an overall incidence rate of 1.829 (95% CI 1.701 – 1.963) aggressive incidents per 100 treatment days, 0.950 incidents with a severity score of ≥ 9 (95% CI 0.859 – 1.049), and 0.606 (95% CI 0.534 – 0.686) attacks against persons, respectively (table 2). The incident rates per bed per year were 7.08 for all incidents, 3.32 for more severe incidents (SOAS-R ≥ 9 points) and 2.35 for physical attacks. On 14.2% (one in 7 days, one per week) of the calendar days within the study period at least one aggressive incident was registered, on 10.0% (1 in 10 days) of the days at least incident with a severity of ≥ 9 and in 7.9% of the days at least one physical attack (one in 13 days, one every two weeks).

**Table 2:** Incidence rates

	n	Rate per 100 treatment days	95% CI	Rate per bed per year	% of calendar days with ≥ 1 incident
All incidents	760	1.829	1.701 – 1.963	7.08	14.2
Incidents with SOAS-R severity ≥ 9	396	0.950	0.859 – 1.049	3.32	10.0
Physical aggression	403	0.970	0.877 – 1.069	3.77	10.3
Purely Verbal aggression	357	0.859	0.772 – 0.953	3.34	9.3
Physical attacks	252	0.606	0.534 – 0.686	2.35	7.9
Incident requiring treatment of the victim	57	0.137	0.104 – 0.178	0.53	2.3

### Severity of aggressive incidents

The severity of all 760 incidents on the SOAS-R severity scale ranged from 0 to 21 with a mean severity of 8.80 ± 4.88 and a median of 9 points. On the VAS, the mean severity rating was 34.40 ± 26.26 with a median of 28 and a range from 0 to 100. There is a significant correlation between these two severity scores of 0.321 (Spearman Rho, p < .001). The correlations were 0.352 in incidents of purely verbal aggression and 0.269 in incidents of physical aggression (table 3).

**Table 3:** SOAS-R- and VAS-Severity-Score

	SOAS-R-Score			VAS			Correlation
	n	Range; Med	Mean ± SD	n	Range; Med	Mean ± SD	r*

All incidents	760	0 – 21; 9	8.8 ± 4.9	669	0 – 100; 28	34.4 ± 26.3	0.321
Physical aggression	403	0 – 21; 10	10.0 ± 5.1	347	0 – 100; 30	36.4 ± 26.7	0.269
Verbal aggression	357	0 – 17; 8	7.5 ± 4.3	322	0 – 100; 27	32.2 ± 25.6	0.352
Physical attacks	252	3 – 21; 11	11.1 ± 5.1	212	0 – 100; 29	36.8 ± 27.9	0.280

* Spearman Rho, all correlations p < 0.001

**Figure 1:**
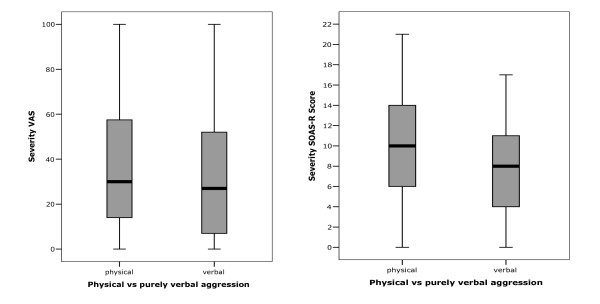
Severity of verbally and physically aggressive incidents (VAS- and SOAS-R-score).

The mean severity of physically aggressive incidents was significantly higher than the severity of purely verbally aggression (Mann-Whitney-U Test p < 0.001 for SOAS-R severity score and p = 0.026 for VAS severity) (figure 1). However, 46% of the purely verbally aggressive incidents had a SOAS-R severity score of ≥ 9 points and were classified as severe incidents, while in contrast 42% of the physically aggressive incidents scored as less severe.

### Incidents followed by a coercive measure

Dependent on their nature and severity, between 43% (verbal aggression) and 67% (SOAS-R-severity score of ≥ 9 points) of the aggressive incidents were followed by a coercive measure, in most cases in form of seclusion or seclusion accompanied by per oral medication or forced injection, while physical restraint was rarely used (table 4).

**Table 4:** Incidents followed by coercive measures

			3 most frequently used forms of coercion in response to the aggressive incidents n (%*)		
Type of incident	n	Followed by coercive measure n (%)	Seclusion	Seclusion + medication p.o.	Seclusion + forced injection

All incidents	760	406 (*53.4%*)	110 (*14.5%*)	96 (*12.6%*)	37 (*4.9%*)
Incidents with SOAS-R severity ≥ 9	396	266 (*67.2%*)	72 (*18.2%*)	64 (*16.2%*)	28 (*7.1%*)
Physical aggression	403	253 (*62.8%*)	71 *17.6%*	53 (*13.2%*)	28 (*6.9%*)
Verbal aggression	357	153 (*42.9%*)	39 (*10.9%*)	43 (*12.0%*)	9 (*2.5%*)
Physical attacks	252	164 (*65.1%*)	55 (*21.8%*)	32 (*12.7%*)	21 (*8.3%*)

* % of all incidents of the respective category

### Proportion of aggressive patients

No aggressive incidents were recorded amongst 1755 (87.0%) of the 2017 patients, while 262 (13.0%) of the patients were responsible for one or more incident, and 6.7% were involved in one or more physical attack. 38 (1.9%) of the patients accounted for 51% of all events, and 58 (2.9%) of the patients accounted for 50% of all attacks (see table 5).

**Table 5:** Percentage of patients involved in aggressive incidents (n = 2017)*

	Patients involved in ...							
	... all incidents		... incidents with SOAS-R-severity ≥ 9		... physical attacks		... incidents requiring treatment of the victim	
	
Incidents per patient	n	%	n	%	n	%	n	%
0	1755	87.0%	1827	90.6%	1881	93.3%	1977	98.0%
1	153	7.6%	130	6.4%	97	4.8%	34	1.7%
2	52	2.6%	70	3.5%	26	1.3%	6	0.3%
3–5	34	1.7%	18	0.9%	10	0.5%		
6–10	16	0.8%	4	0.2%	2	0.1%		
11–20	4	0.2%	3	0.1%	1	0.0%		
>20	3	0.1%						

Total	2017	100.0%	2017	100.0%	2017	100.0%	2017	100.0%

≥ 1 Incidence	262	13.0%	190	9.4%	136	6.7%	40	2.0%

Patients accounting for 50% of incidents	38	1.9%	41	2.0%	58	2.9	3	0.1%

* Calculations based on index episode (one per patient); 636 incidents, 338 severe incidents, 185 physical attacks, 46 incidents requiring treatment

### Associations of patient characteristics with incidence of severe aggressive incidents

The occurrence of one or more aggressive incident was significantly associated with the age of the patient, admission status of the patient, length of stay and diagnostic group (table 6). A lower risk was found in patients aged over 50 (OR 0.67), in patients with a short length of stay (OR 0.46), in patients with a diagnosis of substance abuse (F1) (OR 0.50), and, in patients with neurotic or personality disorders (F4/F6) (OR 0.56). A higher risk was found in involuntary patients (OR 2.16), in patients with a length of stay of ≥ 17 days (OR 2.72), in patients with a diagnosis of schizophrenia (F2) (OR 2.10) or a diagnosis out of the ICD-10 categories F0, F5, F7, F8, F9, F10 (OR 2.16). No significant association with the risk for aggression was found for gender and affective disorders (ICHD-10 F3) (table 6).

**Table 6:** Odds ratios for the occurrence of severe aggressive incidents (n = 2017)

		n	Patients with severe aggressive incident (SOAS-R-Score >8)	Crude odds ratio^#^	
	Factor			OR (95%-CI)	p*

Gender	female	940	8.5%	(ref)	
	male	1077	10.2%	1.21 (0.92–1.60)	0.18
Age^1^	≤ 29 yrs.	537	10.2%	1.04 (0.75–1.43)	0.82
	30–48 yrs.	986	10.2%	(ref)	
	≥ 49 yrs.	493	6.9%	0.67 (0.46–0.99)	0.04
Admission^2^	voluntary	1165	6.6%	(ref)	
	involuntary	746	13.3%	2.16 (1.63–2.90)	<0.001
LOS	≤ 5 days	703	3.7%	0.46 (0.29–0.73)	0.002
	6–16 days	674	7.7%	(ref)	
	≥ 17 days	640	17.5%	2.72 (1.96–3.77)	<0.001
Diagnosis (ICD-10)^†3^	F1	503	5.6%	0.50 (0.33–0.76)	0.001
	F2	590	13.9%	2.10 (1.54–2.88)	<0.001
	F3	336	6.8%	0.68 (0.43–1.08)	0.101
	F4/6	358	5.9%	0.56 (0.35–0.90)	0.017
	Others	136	16.9%	2.16 (1.34–3.48)	0.002

^# ^calculated separately for each variable *wald statistic

^† ^calculated separately for each diagnostic category compared to all other diagnostic groups

^1 ^1 missing ^2 ^106 missing ^3 ^94 missing

### Occurrence of incidents during hospitalisation

One in four of the incidents and 1/3 of the attacks were registered within the first three days, 37.5% within the first 7 days of the hospitalisations and about half of the incidents within the first 14 days (table 7).

**Table 7:** Occurrence of incidents during hospitalisation

	All incidents			Incidents SOAS-R ≥ 9			Physical attacks		
	
	n	%	Cum %	n	%	Cum %	n	%	Cum %
Day of admission	94	12.4	12.4	59	14.9	14.9	46	18.3	18.3
Day 2–3	107	14.1	26.4	56	14.2	29.1	37	14.7	32.9
Day 4–7	84	11.1	37.5	37	9.4	38.5	34	13.5	46.4
Day 8–14	97	12.8	50.3	46	11.6	50.1	27	10.7	57.1
Day 15–30	100	13.2	63.4	56	14.2	64.3	33	13.1	70.2
Day 31–90	146	19.2	82.6	80	20.3	84.6	41	16.3	86.5
Day 91 or later	132	17.4	100.0	61	15.4	100.0	34	13.5	100.0

Total	760	100.0		395	100.0		252	100.0	

Most incidents occurred between 10 a.m. and 8 p.m., with peaks between 10–11 a.m. and 5 to 8 p.m., and they were nearly equally distributed among weekdays, with fewer incidents on Sundays.

### Estimation of underreporting

Shift reports of 191 patients covering 573 treatment days were checked by C.A. and I.N. for descriptions of aggressive incidents and then compared with the SOAS-forms from the respective time-periods and patients. We found 11 incidents not registered on the SOAS form. Two of these incidents included physical aggression (in one event, during a dispute between patients one of these threw a cup against his counterpart; in the other event a patient hit a nurse with a bedpan). Nine of the unreported incidents included only verbal aggression, but only in 2 of theses cases the verbal aggression was explicitly described as threatening and was therefore clearly covered by the definition of aggression included in the SOAS-form. According to our estimation of the severity of these incidents, the severity scores were between 1 and 5 points and none of them would have reached a severity score of 9 ore more. Therefore, calculated for the 41560 treatment days in our study, we would have about 280 unreported incidents less severe physical aggression, but no unreported incidents of a severe nature scoring 9 or more points. Considering our total of 681 reported incidents, this would equal a 30% rate of underreporting of "mild" incidents.

## Discussion

The aim of this study was to describe the frequency and severity of aggressive incidents in acute psychiatric wards in the German speaking part of Switzerland and to explore associations of patients' characteristics with the occurrence of aggressive incidents. The study design aims to meet methodological problems of existing studies.

### Strengths and weaknesses

The inclusion of 24 wards from 12 hospitals and more than 40'000 treatment days makes our study to one of the largest studies addressing aggression specifically in acute wards. Given the close confidence intervals we consider our results as robust and less subject to local variations in single wards or hospitals. To our knowledge this study is the first to report data on a sample of wards representative for a larger geographic area. The strengths of our study include its prospective nature, the use of a standardized and widely used instrument, enabling us to report different incident rates and rates for different types of aggressive incidents, including confidence intervals which are rarely reported. This is one of the few studies including a systematic check of underreporting enabling us to estimate its extent. However, limitations of this study include the observed underreporting of less severe incidents and of patient-to-patient aggression, and the relatively short observation period of 3 month per ward. The participating wards are a convenience sample and therefore we cannot fully exclude the presence of a sampling bias. However, the sample included one out of four acute wards within the study area. Control for differences in the characteristics of participating and non-participating wards showed some differences. Despite of the inclusion criteria for acute admission wards, a limitation related to studies on acute wards is a possible remaining heterogeneity of the wards due to differences in service organisation (e.g. number of beds per catchment area), hospital organisation (e.g. degree of specialisation of wards) and policy (e.g. referrals from admission to other wards). This hampers the generalisability beyond the wards under study. Another limitation is the lack of more detailed information on socio-demographic, clinical and behavioural characteristics of the patients (e.g. data on the severity of psychopathology and illness). The observation periods of the study wards covered all seasons. However, they were unequally distributed and we had more months of observation in autumn and winter than in spring and summer. Thus we cannot exclude a possible seasonal bias.

### Implications and comparison with previous studies

#### Incidence

We found incidence rates per 100 hospitalisation days of 1.8 for all aggressive incidents, of 1 for incidents with a SOAS-R severity score of ≥ 9, and of 0.61 for attacks against persons. On 14% of the calendar days at least one aggressive incident was registered (one per week), on 1 in 10 days at least one more severe incident with a severity of ≥ 9 and in one in 13 days at least one physical attack. These results confirm the findings of studies that aggression is a serious and frequent problem in acute admission wards. Compared to results of the largest studies focusing on acute wards, our overall incidence of 1.8 aggressive incidents per 100 hospitalisation was higher than the rates found in three of these studies, equal to the rate found by Barlow et al [11], but lower that the rate reported by Mellesdal [30] (see table 8). The latter can possibly be explained by the high rate of admissions and occupancy levels up to 133% reported by the authors. However, comparing the rates for physical assaults only, our rates are comparable with others. This finding underlines problems of comparing overall incidence rates. The variance in the incidence rates is lower and there is more overlap in the confidence intervals between the studies when a more narrow definition of aggression is applied. This could possibly be explained by inconsistencies in registering non-physical aggression. E.g. one third of the incidences in our study were physical assaults, compared with 80% found by Grassi et al [29], both using the same instrument (SOAS).

**Table 8:** Comparison of incident rates among 6 larger studies in acute wards

Study	Study size^1^	treatment days^2^	% patients involved	Incident rates per 100 treatment days (95%-CI)	
				All incidents	Assaults
Swiss results	12 h, 24 w	41'560	13%	1.83 (1.70–1.96)	0.61 (0.53–0.69)
Chou et al [28]	4 h, 7 w	56'000	n.a.	1.53 (1.43–1.63)	0.84 (0.77–0.92)
Grassi et al [29]	1 w	27'375^2^	8%	1.20 (1.08–1.34)	0.97 (0.86–1.01)
Barlow et al [11]	1 h, 2 w	18'560^2^	13%	2.05 (1.84–2.27)	0.45 (0.36–0.56)
Mellesdal [30]	1 w	17'430	7%	5.63 (5.28–5.99)	3.15 (2.90–3.43)
Omerov et al [31]	1 h, 2 w	17'400		0.79 (0.66–0.93)	0.60 (0.49–0.72)

^1 ^h = hospitals, w = wards; ^2 ^calculated assuming 95%-occupancy

Among other purposes, data on the incidence of aggression in psychiatry is used to investigate risk factors and as benchmark data in studies on the effectiveness of interventions or services in dealing with aggression [8]. Discrepancies as shown in table 3 add substantially to difficulties in research aimed at reducing aggression and violence.

In our study a small group of 2% of the patients accounted for 50% of all incidents, while no aggressive incidents were registered in 87% of the patients. These findings are consistent with other studies [11,17,30,34,39] and support the demand for a more thorough investigation of this high-risk group [40].

#### Severity

The mean and median severity of all incidents on the SOAS-R severity scale was 9 points. This is supportive for the cut-off of 9 points to separate more and less severe incidents. However, the correlation between the SOAS-R-severity score and the subjective VAS-data was low, lower than the correlations reported by others [16,37] and calling for further investigation on the measurement of severity. Our SOAS-R-severity score of physical assaults of 11.1 ± 5.1 points is comparable to the severity reported by Grassi et al. [29]. 46% of the purely verbally aggressive incidents were classified as severe incidents, while 42% of the physically aggressive incidents scored as less severe. These results underscore the importance to register verbal aggression and not to rely on data restricted to aggression of physical nature.

Corresponding with other studies, we found a small proportion of very severe assaults requiring medical treatment of the victim [11,41].

#### Coercion

Between 43% (verbal aggression), 53% (all events) and 67% (severe incidents) of the aggressive incidents were answered by a coercive measure. This figures are equal to those reported by Nijman et al [42] and Omerov et al [31], where 49% and 46% of the incidents were followed by coercive measures (seclusion with or without restraint and forced injection or physical restraint respectively), but they appear high compared to 28% of incidents followed by parenteral medication or restraints reported by Grassi et al [29].

#### Patients

In our sample a higher risk for the occurrence of aggressive incidents was associated with involuntary admission (OR 2.16; 1.6–2.9), longer length of stay (OR 2.7; 2.0–3.8), F2-diagnoses (OR 2.1; 1.5–2.9) and, other diagnoses than F1, F2, F3. F4/6 (OR 2.1; 1.3–3.5), while this was not the case for age and gender. A higher risk for aggression in involuntary admitted patients and patients with prolonged stay on acute wards has been observed regularly [11,30,42]. However, this finding is not surprising given the fact that danger to others is the main reason for involuntary commitments and one frequent obstacle for discharge. No gender differences in the overall incidence of aggression were found in several studies [11,28,30,42]. However, the relationship of gender to the incidence of aggression appears complex and study results vary according to different types of aggression studied [43]. E.g. findings in some studies include a higher severity of incidents caused by female inpatients [29,30], or higher rates of verbal aggression among women [43]. This was not the case in our sample. In line with others, we found no association of patients' age with a higher risk for the occurrence of aggression [11,30], while younger age was found to be a risk factor in several studies [26,28,29,42]. A higher risk for aggression in patients with a diagnosis of schizophrenia [11,29,30] or psychotic disorder [28] was found in several studies. In contrast, other author's found no associations of diagnostic groups to an increased risk for aggression [42,26,34]. Based on our dataset, the higher risk in our heterogeneous rest-category of other diagnoses (F0, F5, F7, F8, F9, F 10) is difficult to interpret. It may be explained by the patients with organic conditions within this group, as organic mental disorders have been identified as risk factor for violence [30]. However, the utility of diagnoses as risk factor has been questioned by several authors, pointing to the behavioural variation within diagnostic groups and the changes in symptomatology in the course of the illness [30]. In line with this, severity or acuity of illness were identified as more reliable predictors of violence than diagnoses [34].

#### Time after admission

One in four of the incidents and 1/3 of the attacks were registered within the first three days of the hospitalisations and about half of the incidents occurred within the first 14 days. An accumulation of aggressive incidents in the first days after admission has been reported frequently [11,29,30]. However, in contrast to other studies we observed a substantial proportion of incidents later in the course of the hospitalisation. This is an indication not to limit systematic risk assessment to the admission phase.

#### Underreporting

We found a 30% underreporting in mild incidents and no underreporting of more severe incidents scoring 9 or more points. We interpret this as clue to the validity of the SOAS-R in registering serious aggressive incidents. However, the rate of underreporting of less severe incidents including many cases of verbal aggression or aggression directed towards objects leads to an underestimation of the overall aggressivity in acute settings. Furthermore, we cannot completely exclude that some incidents were not registered in the shift reports that we used to control for underreporting on SOAS-R-forms. As shown above, the obvious variability in reporting less severe incidents makes comparisons of total incidence rates problematic. Among the unreported incidents were cases of patient-to-patient aggression, which raises some concern. As the usual reporting-systems requires that staff members are witnesses of an incident, some of the aggression between patients is inevitably undetected, as demonstrated by studies using video surveillance of day rooms [44]. However, there have been complaints of service users on neglecting the negative effects of violence in patients compared to the attention towards victimisation among staff [45].

## Conclusion

Aggressive incidents in acute admission wards are a frequent and serious problem. Due to the large and representative sample size, the prospective study design and the use of a standardized reporting instrument this study, we consider the incidence rates as robust and representative for acute wards in German speaking Switzerland, and thus useful as reference for comparative research and studies aimed at reducing aggression and violence in acute admission wards. Implications for clinical practice include the recommendation to extend the systematic risk assessment beyond the first days after admission. More attention should be given to patient-to-patient aggression. The high rate of coercion as response to aggression underscores the need for research on preventive or less restrictive interventions vis-à-vis aggressive behaviour (see e.g. [46]).

With regard to further research this study supports the call for more in depth research on frequently aggressive patients. It confirms the necessity to differentiate between types of aggressive behaviour when reporting and comparing incidence-data.

## Competing interests

The author(s) declare that they have no competing interests.

## Authors' contributions

The authors have carried out the study together. CA and IN were the principal investigators in this study and collected and organized the data. CA, together with IN and JF, wrote the first draft of the article. JF and IN contributed to the conception of the study and to the statistical analysis and interpretation of the data. TD, RH and HJH contributed to the conception of the study and revised the manuscript critically. All authors read and approved the manuscript.
